# Predictors of early mortality and effectiveness of antiretroviral therapy in TB-HIV patients from Brazil

**DOI:** 10.1371/journal.pone.0217014

**Published:** 2019-06-06

**Authors:** Fernanda O. Demitto, Carolina A. S. Schmaltz, Flávia M. Sant’Anna, María B. Arriaga, Bruno B. Andrade, Valeria C. Rolla

**Affiliations:** 1 Programa de Pós-Graduação em Pesquisa Clínica em Doenças Infecciosas, Instituto Nacional de Infectologia Evandro Chagas, Fundação Oswaldo Cruz, Rio de Janeiro, Brazil; 2 Laboratório de Pesquisa Clínica em Micobacterioses (LAPCLIN-TB), Instituto Nacional de Infectologia Evandro Chagas, Fundação Oswaldo Cruz, Rio de Janeiro, Brazil; 3 Multinational Organization Network Sponsoring Translational and Epidemiological Research (MONSTER) Initiative, Salvador, Brazil; 4 Instituto Gonçalo Moniz, Fundação Oswaldo Cruz, Salvador, Brazil; 5 Escola Bahiana de Medicina e Saúde Pública (EBMSP), Salvador, Brazil; 6 Universidade Salvador (UNIFACS), Laureate Universities, Salvador, Brazil; 7 Wellcome Centre for Infectious Disease Research in Africa, Institute of Infectious Disease and Molecular Medicine, University of Cape Town, Cape Town, Republic of South Africa; 8 Division of Infectious Diseases, Department of Medicine, Vanderbilt University School of Medicine, Nashville, TN, United States of America; National Institute for Infectious Diseases IRCCS Lazzaro Spallanzani, ITALY

## Abstract

**Background:**

The implementation of antiretroviral (ARV) therapy caused a significant decrease in HIV-associated mortality worldwide. Nevertheless, mortality is still high among people living with HIV/AIDS and tuberculosis (TB). ARV-naïve HIV patients coinfected with tuberculosis (TB) have more options to treat both diseases concomitantly. Nevertheless, some TB-HIV patients undertaking ARVs (ARV-experienced) are already failing the first line efavirenz-based regimen and seem to display different response to second line ARV therapy and exhibit other predictors of mortality.

**Methods:**

We performed a retrospective cohort study including 273 patients diagnosed with TB-HIV and treated at a referral center in Rio de Janeiro, Brazil, between 2008 and 2016. Multivariate analysis and Cox regression models were used to evaluate the effectiveness of ARV therapy regimens (viral load [VL] <80 copies from the 4^th^ to 10^th^ months after TB therapy introduction) and to identify predictors of early mortality (100 days after TB therapy initiation) considering ARV-naïve and ARV-experienced patients adjusting for sociodemographic, clinical and therapeutic covariates.

**Findings:**

Survival analysis included 273 patients, out of whom 154 (56.4%) were ARV-naïve and 119 (43.6%) were ARV-experienced. Seven deaths occurred within 6 months of anti-TB treatment, 4 in ARV-naïve and 3 in ARV-experienced patients. Multivariate analysis revealed that in ARV-naïve patients, the chance of death was substantially higher in patients who developed immune reconstitution inflammatory syndrome during the study follow up (HR = 40.6, p<0.01). For ARV-experienced patients, similar analyses failed to identify factors significantly associated with mortality. Variables independently associated with treatment failure for the ARV-naïve group were previous TB (adjusted OR [aOR] = 6.1 p = 0.03) and alcohol abuse (aOR = 3.7 p = 0.01). For ARV-experienced patients, a ritonavir boosted. Protease Inhibitor-based regimen resulted in a 2.6 times higher risk of treatment failure compared to the use of efavirenz based ARV regimens (p = 0.03) and High baseline HIV VL (p = 0.03) were predictors of treatment failure.

**Conclusions:**

Risk factors for mortality and ARV failure were different for ARV-naïve and ARV-experienced patients. The latter patient group should be targeted for trials with less toxic and rifampicin-compatible drugs to improve TB-HIV treatment outcomes and prevent death.

## Introduction

Antiretroviral (ARV) therapy was one of the greatest achievements in medicine of the last decade due to the significant decrease in HIV-associated mortality, most significantly observed in low and high-developed countries [1,2]. Since 1986, the Brazilian Ministry of Health offers antiretrovirals free of charge, as well as assessment of CD4^+^ lymphocyte counts, HIV viral load (VL) and more recently genotyping, to all patients in the public health system [3]. However, although this policy has been implemented in Brazil and other high burden of tuberculosis (TB) countries, mortality is still high among people living with HIV/AIDS and TB. One of the reported reasons for this scenario is the loss of follow up of patients after diagnosis of HIV infection, with patients being reluctant to initiate ARV due to misinformation and/or awareness about the benefits of this therapy [4] in addition to toxicity of TB-HIV concomitant therapy [5].

In 2009, Sant’Anna et al. conducted a study in TB-HIV patients to evaluate the HIV VL control after ARV therapy implementation in ARV-naïve (those persons who have never received ARV before) and ARV-experienced patients (those who have used ARV regimens previously) from Rio de Janeiro, Brazil [6]. The authors found that, for ARV-naïve patients, the best results were achieved with efavirenz-based regimens. However, for ARV-experienced patients, the effectiveness was lower than that found in naïve patients, and efavirenz based regimens were not effective, which was attributed to probable acquired drug resistance [6]. Additional studies in this population revealed that ARV regimens containing a Protease Inhibitor (PI) boosted with ritonavir were associated with better virologic control but also linked to increased incidence of severe adverse reactions [7]. Recently, Rifabutin incorporation in the Brazilian HIV program [8] brought some progress in TB-HIV treatment, increasing the possibilities of concomitant ARV regimens. ARV-experienced patients have a few options of effective ARV drugs that usually cannot be used with rifampicin. Therefore, treatment outcomes in ARV-experienced patients receiving therapy for TB and AIDS could be improved with Rifabutin [8]. Moreover, new ARVs were incorporated in the Brazilian HIV guidelines, such as the PI Darunavir and a new class of Integrase Inhibitors (II) (Raltegravir), which although not yet broadly used, could also positively influence ARV effectiveness [9].

Another study conducted by our group [10] has previously shown that the predictors of early mortality in ARV-naïve or ARV-experienced patients with TB diagnosis appear to be different. Among ARV-naïve patients, mortality was influenced by TB severity and no ARV use during TB treatment, possibly because of a late presentation for medical assistance. For ARV-experienced patients, delays in TB diagnosis for more than 120 days as well as poor control of viral replication were associated with higher mortality rates [10].

Although several previous studies have explored different aspects of clinical and pharmacological management of TB-HIV patients, scarce data is available on effectiveness of ARV therapy in patients undergoing TB treatment, and mainly in ARV-experienced patients, especially in Brazil. Since more ARVs are becoming available in most of the TB high burden countries, the number of patients with TB who will be already on ARV therapy might increase. Therefore, it is critical to evaluate the effectiveness of ARV regimens used during TB treatment and risk factors associated with early mortality in TB-HIV patients, not only in ARV-naïve but also considering previous use of ARVs. Although no differences in mortality rate has been shown so far between ARV-naïve and ARV-experienced patients, different predictors of mortality may drive implementation of distinct approaches in clinical management of these groups. In the present study, we explored this matter in a population from a high burden area of TB-HIV in Brazil.

## Methods

### Ethics statement

The study was approved by the Institutional Review Board of the Instituto Nacional de Infectologia Evandro Chagas (CAAE: 71191417.8.0000.5262). Written informed consent was obtained from all participants, and all clinical investigations were conducted according to the principles expressed in the Declaration of Helsinki.

### Population and design

A prospective cohort has been followed at the Clinical Research Laboratory on Mycobacteria (LAPCLIN-TB) of the Instituto Nacional de Infectologia (INI) Evandro Chagas, Fundação Oswaldo Cruz, Rio de Janeiro, Brazil, since 2000. The present study is a retrospective assessment performed between 2008 and 2016, with data obtained from this cohort. Data were collected from electronic medical records based on standardized information of a defined template used in each patient’s visit for the whole cohort. Patients with 18 years and older, HIV-seropositive, with clinical signs and symptoms of TB with or without a positive culture of any site (pulmonary or extrapulmonary) were included. For those who had a negative culture, a positive therapeutic test with TB drugs was considered, after excluding other opportunistic diseases for differential diagnosis. Patients that initiated TB treatment and were diagnosed later with Non-tuberculous Mycobacteria as well as those who showed rifampicin and isoniazid resistance (multidrug resistance) were excluded.

### Definitions

TB was classified as pleuropulmonary (when restricted to the lungs and/or pleura), extrapulmonary (when just one extra-pulmonary site was identified) or disseminated (involving spleen, liver, bone marrow, or at least 2 noncontiguous sites) [11].

Comorbidity was considered when two or more diseases were simultaneously detected in the same person, such as diabetes, chronic obstructive pulmonary disease, hepatitis B or C, hypertension, amongst others. ARV-naïve patients were defined as those who had not received ARVs before TB diagnosis whereas ARV-experienced patients were those who were undertaking ARVs or had already been treated with ARVs at TB diagnosis.

TB-associated immune reconstitution inflammatory syndrome (IRIS) was defined as a documented worsening of TB signs or symptoms during antituberculous treatment and following the initiation of ARV, not explained by any other diseases or by an adverse effect of drug therapy, as described previously [12,13]. Suggestive histopathologic findings of a lymph node biopsy and/or an improvement of CD4^+^ cell counts after appearance of the clinical signs of IRIS corroborated the diagnosis in some patients. If a biopsy was not available, lymph node enlargement with inflammatory signs temporally related with ARV introduction was considered IRIS.

The cause of death was determined after thorough review of relevant clinical, microbiological and pathological data of each deceased patient. Only deaths due to TB were analyzed. Early deaths were defined as deaths in the first 100 days of TB therapy. ARV effectiveness was defined as HIV VL ≤ 80 copies/mL between months 4 to 10 after TB therapy initiation.

### TB diagnosis and follow up visits

Visits included in this study were done at baseline (TB diagnosis and treatment initiation), 15 days and 30, 60, 90, 120 e 180 days after TB therapy initiation. ARV were initiated after TB treatment according to decision from each physician and following the Brazilian TB treatment Guidelines [14].

Information collected at the baseline visit included sociodemographic data as well as previous TB and ARV treatments, clinical presentation of TB, comorbidities like diabetes, hypertension, hepatitis (B and C), opportunistic diseases as well as CD4 counts and HIV VL among other variables.

### Antituberculous and antiretroviral therapy

ARV therapy was offered according to contemporary Brazilian National Guidelines that were periodically updated [14].

The first line antituberculous regimen was the combination of rifampicin, isoniazid and pyrazinamide during the two initial months, followed by rifampicin and isoniazid during four months, except when the continuation phase needed to be extended to seven months such as in cases with central nervous system TB. From July 2009 on, ethambutol was added to the intensive phase regimen following a new recommendation of the National TB program of the Brazilian Ministry of Health [15]. TB treatment was adjusted in cases of severe adverse reactions, drug resistance and ARV regimens that precluded the use of rifampicin.

### Statistical analysis

Descriptive statistics was used to present data, which was presented as proportions, mean ± standard deviation (SD), or median and interquartile range (IQR), depending on Gaussian distribution assessed by the Kolmogorov-Smirvov test. The Fisher’s and Chi-square tests were used to compare categorical variables between study groups. Continuous variables were compared using the Mann-Whitney *U* test. Bivariate analysis was used to describe the association with socio-demographic and clinical variables from baseline exposure to ARV. The first outcome was assessed based on the following status: patients who did not die from TB had their follow-up censored on the 100^th^ day after the start of antituberculous therapy. Overall survival was assessed using the Kaplan-Meier (KM) method. We used Cox proportional hazards regression models to estimate hazard ratios (HRs) and 95% confidence intervals (CIs). A multivariate regression model was used to evaluate the effectiveness of ARV considering ARV-naïve and ARV-experienced. Variables with univariate p-value ≤ 0.2 were selected to the multivariate model to assess the odds ratios (OR). The analyses were performed using SPSS version 24.0 software package. All analyses were pre-specified. Differences with two-tailed p-values <0.05 were considered statistically significant. Raw data is depicted in S1 File.

## Results

### Cohort description

During the period from 2008 to 2016, 285 patients were screened, 11 patients were excluded because they were diagnosed with atypical Mycobacteria and one with multidrug resistant TB.

Survival analysis included 273 patients, out of whom 154 (56.4%) were ARV-naïve and 119 (43.6%) were ARV-experienced. Seven early deaths were observed, 4 in ARV-naïve patients and 3 in ARV-experienced. There were 12 cases of TB-associated IRIS, among which 4 (33.34%) were pulmonary, 1 (8.33%) was extrapulmonary and 7 (58.33%) were disseminated TB. Eight (5.2%) ARV-naïve and 4 (3.4%) ARV-experienced patients developed IRIS (Table 1). Eleven IRIS cases occurred in patients using nucleoside reverse transcriptase inhibitors (NRTI) + non-nucleoside reverse transcriptase inhibitors (NNRTI) and 2 cases in those who used NRTI +PI during TB treatment. None of the IRIS cases had resistance to first line TB drugs. For the effectiveness analysis, 186 patients with HIV VL results available at the end of TB treatment were included, 116 (62.4%) were ARV-naïve and 70 (37.6%) were ARV-experienced. By the end of follow up, 127 (68.3%) patients had VL ≤ 80 copies/mL, with 87 (68.5%) in the ARV-naïve group and 40 (31.5%) in the ARV-experienced group. In addition, 59 (31.7%) had VL > 80 copies/mL, 29 (49.2%) in the ARV-naïve group and 30 (50.8%) in the ARV-experienced. Thus, effectiveness of ARV therapy was significantly higher in the group of ARV-naïve individuals (odds ratio [OR] 2.1, 95% CI: 1.1–4.0, p = 0.02).

**Table 1:** Baseline characteristics of the study participants stratified according to exposure to antiretroviral therapy at enrollment.

Characteristic	Study population (N = 273)		p-value
	ARV-naïve	ARV-experienced	
	n = 154 (56.4%)	n = 119 (43.6%)	
Male	114 (74.0)	81 (68.1)	0.3
Race: white	69 (44.8)	36 (30.3)	0.1
Age z 40 years	67 (43.5)	52 (43.7)	1.0
Educational level			1.0
*≥ 5 years of schooling*	93 (60.4)	72 (60.5)	
*< 5 years of schooling*	60 (39.6)	47 (39.5)	
Family income			0.7
*> 2 Minimal wage*	26 (18.2)	17 (16.0)	
*≤ 2 Minimal wage*	117 (81.8)	89 (84.0)	
Not married status	105 (68.2)	81 (68.1)	1.0
Homo/bisexual	54 (35.5)	36 (30.3)	0.4
Smoker	80 (52.3)	62 (53.4)	0.9
Alcohol abuse	48 (31.6)	41 (34.5)	0.7
Use of illicit drugs	40 (26.1)	37 (31.4)	0.3
Comorbidity	44 (28.6)	50 (42.0)	0.02
Viral hepatitis (B or C)	12 (8.4)	14 (12.0)	0.4
Weight loss (>10%)	130 (85.0)	75 (63.0)	<0.01
Previous tuberculosis	11 (7.1)	54 (45.4)	<0.01
Positive hemoculture for *M*. *tuberculosis*	14 (15.6)	5 (5.8)	0.05
Resistant to any TB drug	19 (22.1)	7 (10.9)	0.08
Baseline VL ≥ 5 log	95 (70.4)	28 (29.2)	<0.01
Clinical form of tuberculosis			0.006
*Pleuropulmonary*	79 (51.3)	76 (63.9)	
*Extrapulmonary*	15 (9.7)	18 (15.1)	
*Disseminated*	60 (39.0)	25 (21.0)	
Baseline CD4^+^ cell count	169 (129–195)	205 (134–278)	0.17
median (IQR)			
Paradoxical Reaction (IRIS)			0.56
*Yes*	8 (5.2)	4 (3.4)	
*No*	145 (94.8)	114 (96.6)	
ARV use before TB			-
*NRTI+NNRTI*	NA	39(32.8)	
*NRTI+PI*	NA	35 (29.4)	
*NRTI+II*	NA	2 (1.7)	
*NRTI +PI+II*	NA	1 (0.8)	
*NRTI*	NA	1 (0.8)	
ARV use after TB			<0.01
*NRTI+NNRTI*	125 (94.0)	47 (42.0)	
*NRTI+PI*	7 (5.3)	57 (50.9)	
*NRTI+II*	1 (0.8)	4 (3.6)	
*NRTI +PI+II*	0 (0.0)	3 (2.7)	
*NRTI*	0 (0.0)	1 (0.9)	

p-value based on Chi-squared test; NA, nonapplicable; ARV = antiretroviral

NRTI = nucleoside reverse transcriptase inhibitors; NNRTI = non-nucleoside reverse transcriptase inhibitors; PI = protease inhibitors; II = integrase inhibitors

Baseline characteristics of both groups are shown in Table 1. Comorbidities and previous TB were more frequent in the ARV-experienced group, while loss of more than 10% of body weight, HIV VL ≥5 log and disseminated clinical forms of TB were more frequent in the ARV-naïve group. Positive blood cultures were more frequent in the ARV-naïve group while ARV-experienced individuals exhibited a trend to have more monoresistance to TB drugs.

In ARV-naïve patients, the univariate model (Table 2) showed that the risk of dying was higher in patients who developed IRIS after commencement of ARV therapy (hazard ratio [HR] = 6.8, 95% CI: 1.3–35.1, p = 0.005). The median time between onset of ARV and time at which IRIS occurred was 84 days (IQR 27–152). The median time between ARV onset and HIV VL drop before IRIS onset was 50.5 days (IQR 16-5-249). In addition, increases of 1 log in baseline HIV VL values had a tendency to exhibit augmented risk of early death (≤ 100 days after therapy initiation) as well, however, without reaching statistical significance (Table 2). A multivariate regression analysis model confirmed association between occurrence or IRIS and increased risk of early death (Table 2). For ARV-experienced patients, univariate and multivariate analyses failed to reveal risk factors significantly associated with early death (Table 3).

**Table 2:** Cox analysis for early mortality (100 days) for ARV-naïve patients.

Variable	Univariate analysis			Multivariate analysis		
	HR	95% CI	p-value	HR adjusted	95% CI	p-value
IRIS	6.8	(1.3–35.1)	0.005	40.6	(5.1–320.8)	<0.01
Baseline HIV VL (log10)	3.6	(0.8–16.8)	0.11	5.4	(0.9–33.0)	0.07

Only IRIS and baseline HIV VL had univariate p-values ≤ 0.2 and thus were selected to be used in the multivariate model. HR: Hazard ratio; CI: confidence interval; IRIS = Paradoxical reaction

**Table 3:** Cox analysis for early mortality (100 days) for ARV-experienced patients.

Variable	Univariate analysis			Multivariate analysis		
	HR	95% CI	p-value	HR adjusted	95% CI	p-value
Age (years)	1.1	(0.9–1.2)	0.08	1.1	(1.0–1.3)	0.09
Extrapulmonary TB	6.6	(0.9–47.2)	0.06	2.8	(0.2–35.3)	0.43
Baseline HIV VL ≥ 5 log	4.7	(0.4–51.6)	0.11	6.5	(0.5–86.3)	0.16

Only age, extrapulmonary TB and baseline HIV VL ≥5 log had univariate p-values ≤ 0.2 and thus were selected to be used in the multivariate model. HR: Hazard ratio; CI: confidence interval

### ARV effectiveness

The response to ARV were analyzed considering if patients were ARV-naïve or ARV-experienced. In univariate Cox regression model (Table 4) alcohol abuse (p<0.01), viral hepatitis (p = 0.02) and previous TB (p = 0.02) were factors associated with ARV failure for the ARV-naïve group. In the multivariate model, variables associated with a worse effectiveness were previous TB (p = 0.03), alcohol abuse (p = 0.01) and a borderline significance for viral hepatitis (p = 0.06).

**Table 4:** Factors associated with antiretroviral therapy failure for ARV-naïve patients.

Variable	Univariate analysis			Multivariate analysis		
	OR	95% CI	p-value	OR adjusted	95% CI	p-value
Alcohol abuse	5.2	(2.1–12.8)	<0.01	3.7	(1.3–10.0)	0.01
Viral hepatitis (B or C)	6.2	(1.4–28.0)	0.02	5.0	(1.0–25.9)	0.06
Previous tuberculosis	5.8	(1.3–26.2)	0.02	6.1	(1.2–31.3)	0.03

OR: Odds ratio; CIs: confidence intervals

The risks factors associated with treatment failure for ARV-experienced patients in the univariate analysis were baseline HIV VL (log10) and ARV treatment with PI-based regimens. In the multivariate analysis, patients treated with a ritonavir boosted PI had 2.6 times higher risk of treatment failure compared to the use of other regimens and the other factor associated with treatment failure was baseline VL (log10). No other risk factors were identified in both groups (Table 5).

**Table 5:** Factors associated with ARV failure for ARV-experienced patients.

Variable	Univariate analysis			Multivariate analysis		
	OR	95% CI	p-value	OR adjusted	95% CI	p-value
Baseline HIV VL (log10)	1.6	(1.1–2.2)	0.009	1.5	(1.0–2.1)	0.03
Protease Inhibitor based regimens	4.9	(1.7–13.8)	0.003	3.6	(1.1–11.1)	0.03

OR = Odds ratio; CI = confidence intervals; VL = viral load

## Discussion

Tuberculosis in HIV-infected patients is still a challenge due to a significant number of deaths concentrated in the first months of TB treatment, toxicity and interactions of TB and ARV drugs in addition to IRIS, which has been described worldwide [16].

In our study, ARV-naïve and ARV-experienced patients were different with regard to several characteristics. ARV-naïve patients had more B or C hepatitis, higher HIV VL at baseline, more disseminated TB with positive blood cultures, significant weight loss and more TB drug resistance. ARV-experienced patients had more frequently previous TB and exhibited more comorbidities. Furthermore, additional analyses on early survival demonstrated that IRIS and high baseline HIV VL were risk factors for early mortality in ARV-naïve but not for ARV-experienced patients. Similarly to our results, the Camelia study [17] reported that even with early implementation of ARV therapy, no deaths associated with IRIS were observed, including in ARV-naïve TB-HIV patients. In addition, lack of ARV therapy in TB-HIV patients was a risk factor in previous study [10] that was not detected in our recent analysis, probably due to the extensive use of ARV in our current cohort.

In previous studies from our group, a delay of more than 120 days from presentation of symptoms to TB diagnosis was linked to increased early mortality of ARV-experienced patients [10]. Such delay occurred in circumstances where a patient had a negative screening for acid-fast bacilli in sputum smears and the assistant physician decided to wait for a positive mycobacterial culture to start TB treatment, for example [10]. In recent years, Xpert -MTB-RIF has become available and a rapid TB screening has been done more frequently [18]; this could have contributed to a faster TB diagnosis in patients living with HIV, decreasing the impact of late TB treatment start in these patients mortality although the median time to diagnosis have not changed.

Noteworthy, our study revealed that occurrence of IRIS was an important risk factor for mortality in ARV-naïve patients, although in previous years such relationship had not been found [10]. In Brazil, a low incidence of IRIS in TB-HIV patients has been reported [19]. We have been working to better understand this phenomenon but the incidence rate have not changed in recent years in our site [20], differently from other countries in the south hemisphere with rates ranging from 22 to 36% [21–23].

Herein, we also analyzed the risk factors for poor response to ARV after TB diagnosis and, again, they were distinct for the two groups of patients differentiated based on ARV exposure. It is possible that there was a higher risk of hepatotoxicity associated with anti-TB and ARV drugs in patients already with alcohol or viral-induced hepatic injury. For ARV-experienced patients, a higher risk of treatment failure was observed in those with higher baseline HIV VL values and those treated with PIs, in other words, those patients with resistance to first line ARV (efavirenz-based regimens). For that population, the options are use lopinavir boosted with ritonavir (800–200 mg or 400–400 mg) and keep the anti-TB drugs in a fixed dose [24,25] or deconstruction of TB fixed dose combination to use Rifabutin (for those who have it available in the public health units) concomitant to others PIs [8]. Our previous study also revealed that patients treated with PI (in both situations described above) had 3.08 times more risk of treatment failure compared with patients using efavirenz-based ARV regimens [6]. Ritonavir boosted PI regimens are toxic and when added to antitubercular drugs may result in interruption of both therapies; no other ARV regimen compatible with rifampicin is recommended in Brazil for instance.

However, some patients develop TB using efavirenz with undetectable HIV VL, and if it turns out to be a case which is sensitive to this drug, one could keep the same regimen and have lower risk of treatment failure. A new class of ARV, the integrase inhibitors, is being used in some countries and was recently incorporated in Brazil [9]. Raltegravir has been recently studied in an *Agence Nationale de Rechèrche sur le SIDA* (ANRS) clinical trial, which demonstrated that a regimen including this drug is as effective as efavirenz based regimens to treat TB-HIV ARV-naïve patients [26]. Raltegravir is a very well tolerated drug but has a low genetic barrier (needs few mutations to acquire resistance) and should not be used in patients with resistance to efavirenz. Another integrase inhibitor recently introduced in Brazil was Dolutegravir [9], which has a good genetic barrier and could be the treatment of choice for TB-HIV patients with resistance to efavirenz. A recent trial reported promising results in naïve patients with very few adverse reactions [27], this drug was already recommended by the WHO, recently [16]. We urgently need better therapies for TB-HIV patients to improve treatment effectiveness and survival. ARV-experienced patients should be a focus of new studies as well as identification of risk factors for mortality and treatment failure to allow effective interventions.

Our study had some limitations, such as the low number of participants in each group and the proportion of patients for whom HIV VL results were available to achieve effectiveness of ARV. Moreover, adherence to treatment was self-reported, and we have not considered due to the low level of reliability. If patients had not taken their drugs correctly, effectiveness of ARVs could have been harmed and we would not have been able to annotate reliably. Another limitation was the low number of IRIS cases that was not enough to explore subgroup analyzes. Regardless, the present study adds to the current knowledge in the field TB-HIV therapies as it demonstrates that risk factors for mortality and ARV failure were different for ARV-naïve and ARV-experienced patients and latter group should be targeted for trials with less toxic rifampicin-compatible drugs to improve TB-HIV treatment outcomes in high burden TB and HIV countries.

## Supporting information

S1 File Raw data used for the analyzes. (XLSX)
